# Cladribine Treatment Improved Homocysteine Metabolism and Increased Total Serum Antioxidant Activity in Secondary Progressive Multiple Sclerosis Patients

**DOI:** 10.1155/2020/1654754

**Published:** 2020-03-14

**Authors:** Anna Jamroz-Wiśniewska, Jerzy Bełtowski, Grażyna Wójcicka, Halina Bartosik-Psujek, Konrad Rejdak

**Affiliations:** ^1^Department of Neurology of Lublin Medical University, Lublin, Poland; ^2^Department of Pathophysiology of Lublin Medical University, Lublin, Poland; ^3^Department of Neurology of Rzeszow University, Rzeszow, Poland

## Abstract

Hyperhomocysteinemia plays a crucial role in the pathogenesis of many diseases of the central nervous system (CNS). The nervous system is particularly sensitive to high homocysteine (Hcy) level mainly due to its prooxidative and cytotoxic effects. Cladribine, a drug recently registered for the treatment of multiple sclerosis (MS), possesses additionally neuroprotective effects which are independent of its peripheral immunosuppressant action. Accumulating evidence suggests that oxidative stress and homocysteine thiolactone-mediated protein homocysteinylation play a causal role in MS. Both of these processes may be attenuated by paraoxonase 1 (PON1). Therefore, in the present study, we aimed to examine whether the beneficial effects of the drug in MS patients with a secondary progressive (SP) clinical course, treated with cladribine subcutaneously (s.c.), may be related to its ability to modify serum PON1 activity, Hcy concentration, and protein homocysteinylation, as well as to correct total antioxidant status. A total of 118 subjects were enrolled into the study: (1) patients with a SP type of MS, SP-MS (*n* = 40); (2) patients with a relapsing-remitting (RR) type of MS, RR-MS (*n* = 30); and (3) healthy people (*n* = 48). Patients with SP-MS were treated with cladribine. The drug was given in SP-SM patients s.c. six times every 6 weeks up to a total mean cumulative dose of 1.8 mg/kg. PON1 activity was assessed spectrophotometrically. The level of Hcy, homocysteine thiolactone (HTL) attached to plasma proteins (N-Hcy-protein), and antibodies against homocysteinylated proteins was assessed with an enzyme immunoassay. The total antioxidant activity of the serum was assessed with the ferric-reducing activity of plasma (FRAP) method. Basically, there was no difference in PON1 activity between untreated SP-MS, RR-MS, and control subjects. Serum Hcy was significantly higher in RR-MS patients (*p* < 0.001) and in SP-MS patients (*p* < 0.01) compared to the control group. The N-Hcy protein level was higher in RR-MS patients (*p* < 0.05) in comparison to the control group. Moreover, the elevated level of antibodies against homocysteinylated proteins was observed in the serum of patients with SP-MS. The total antioxidant capacity of serum was lower in MS patients vs. the control group (*p* < 0.001). After cladribine treatment, the activity of PON1 did not change in SP-MS patients, whereas cladribine treatment decreased the level of total Hcy (*p* < 0.05). Treatment with cladribine increased the total serum antioxidant activity in SP-MS patients (*p* < 0.01). The Expanded Disability Status Scale (EDSS) score did not change in SP-MS patients. Cladribine treatment in the SP-MS group attenuates hyperhomocysteinemia-induced protein homocysteinylation (n.s.). It also stabilises the neurological condition of SP-MS patients. The stabilisation of a neurological condition observed in SP-MS patients after cladribine treatment may be partially related to its ability to reduce elevated Hcy level and to improve serum antioxidant potential.

## 1. Introduction

Multiple sclerosis (MS) is a chronic inflammatory and neurodegenerative disease that affects predominantly young people. It is associated with the development of disability and shortening of mean lifetime by about 10 years. The etiopathogenesis of MS is still under investigation, and the role of oxidative stress is examined. The development of inflammation and demyelination in MS is caused by the activation of the central nervous system (CNS) immune cells that leads to the production of reactive oxygen species (ROS) and lipid peroxidation [1].

Recently, a new drug, cladribine, has been launched in an oral form to the pharmaceutical market. It has been registered by both FDA (Food and Drug Administration) and EMA (European Medicines Agency) for the treatment of a relapsing-remitting type of MS (RR-MS) on the basis of CLARITY (CLAdRIbine Tablets treating multiple sclerosis orallY) study [2] and by FDA for the treatment of an active secondary progressive type of MS (SP-MS) (https://www.fda.gov). Previously, cladribine was used with good results in a parenteral form in phase II trials in progressive MS patients [3]. It is a purine analogue, 2-chloro-2′-deoxyadenosine, 2Cda (C_10_H_12_CIN_5_O_3_), that was synthesized in the early 1980s and originally licensed as a chemotherapeutic agent to treat hairy cell leukaemia; it is used also in B cell chronic lymphocytic leukaemia. In its subcutaneous (s.c.) form, it is bioavailable in 100% comparing to 42% of oral drug [4, 5].

In the cells, cladribine molecule is phosphorylated by deoxycytidine kinase to 2-chloro-2′-deoxy-*β*-D-adenosine monophosphate that is resistant to deamination by adenosine deaminase. This metabolite accumulates intracellularly in lymphocytes and monocytes because of a low level of deoxynucleotide deaminase in these cells. It is then converted into the active form, triphosphate deoxynucleotide, leading to cell death. As a result, there is prolonged depletion predominantly in circulating B lymphocytes [5].

Cladribine leads to depletion of deoxyadenosine and imbalance in deoxythymidine, deoxyguanidine, and deoxycytidine. The drug penetrates to the CNS [6] and may potentially influence the level of oxidative stress due to possible cytotoxic effects [6, 7].

Paraoxonase 1 (PON1) is an esterase that was first described for its capacity to hydrolyse organophosphates and pesticides. However, PON1 basically is a lactonase and the ability to hydrolyse organic phosphates (paraoxon) and aromatic esters (phenyl acetate) is its additional activity. In addition, PON1 possesses peroxidase activity and reduces lipid peroxides generated in cell membranes and lipoproteins in the setting of oxidative stress [8].

PON1 circulates in the blood attached to high-density lipoproteins (HDL) and is also present in minor quantities in the plasma membranes. As a lipoprotein-associated enzyme, PON1 contributes to antioxidant and anti-inflammatory properties of HDL protecting low-density lipoproteins (LDL) against oxidative modification and degrading proinflammatory products within oxidized LDL (ox-LDL). Interestingly, in MS patients, the positive correlation between the level of ox-LDL and clinical activity of the disease has been found [9].

The natural substrate of PON1 is homocysteine thiolactone (HTL), the toxic endogenous metabolite of homocysteine (Hcy) which contributes to detrimental effects of this amino acid [10, 11]. HTL spontaneously reacts with *ɛ*-NH_2_ (amino) groups of protein lysine residues (protein N-homocysteinylation) leading to their damage. In addition, N-homocysteinylated proteins induce the humoral immune response [7, 11]. This is why, by breaking down HTL, PON1 protects against N-homocysteinylation [10, 11]. There are two common polymorphisms of PON1 that lead to glutamine–arginine substitution at 192 (Q192R) and a leucine–methionine substitution at 55 (L55M). They are associated with a variety of diseases, including Parkinson's disease, Alzheimer's disease, stroke, ischemic heart disease, familial hypercholesterolemia, and diabetes mellitus type 2 [7, 12]. Also, low plasma PON1 activity has been documented in patients with CNS diseases, e.g., MS or Parkinson's disease [13]. As cladribine may increase the level of oxidative stress, we studied the activity of PON1 in patients treated with cladribine.

Hyperhomocysteinemia has been associated with some neurological diseases such as Alzheimer's and Parkinson's diseases [14]. Recently, we observed that both total Hcy (tHcy) level and homocysteinylation of plasma proteins are increased in MS patients [15]. Similar results were obtained in the other studies [14, 16, 17]. It has been suggested that hyperhomocysteinemia can play a role in the pathogenesis of MS. Through homocysteinylation of proteins, hyperhomocysteinemia can induce immune process leading to demyelination and inflammation of the cells in the CNS of MS patients. The results of a study by Ramsaransing et al. [18] confirm that hyperhomocysteinemia is not associated with the progression of MS suggesting that it is rather bound to inflammatory processes in the CNS in MS.

However, it is unclear if hyperhomocysteinemia is affected by the respective pharmacotherapy. Still, it is known that cladribine inhibits S-adenosylhomocysteine (SAH) hydrolase through adenosine deaminase inhibition. Adenosine accumulation inhibits SAH hydrolase resulting in nicotinamide adenine dinucleotide (NAD) depletion and adenosine triphosphate (ATP) depletion. Accumulation of SAH inhibits deoxyribonucleic acid (DNA) methyltransferase leading to Hcy depletion [19]. As far as we know, the effect of cladribine on circulating PON1 activity and its ability to protect plasma protein against homocysteinylation in MS have not yet been evaluated.

The aim of this study was to assess the effect of cladribine on PON1 activity, tHcy concentration, and protein N-homocysteinylation in the serum of patients with SP-MS.

## 2. Patients and Methods

### 2.1. Patients and Controls

A total number of 118 subjects hospitalized in the Department of Neurology of Lublin Medical University were involved in this study: patients with a SP type of MS (*n* = 40), patients with a RR type of MS (*n* = 30), and healthy individuals (*n* = 48). All individuals agreed to participate in the study. MS patients were diagnosed with clinically definite MS according to 2010 revisions to McDonald criteria [20]. SP-MS patients with an increase of disability in the Expanded Disability Status Scale (EDSS) score of 2 points or more during the last 2 years [21] were qualified to the treatment with cladribine. All patients from the SP-MS group received cladribine. These patients were not treated with any other immunosuppressive or immunomodulating drug during the last 2 years. RR-MS patients were included into the study before beginning of the treatment without any immunomodulating drug. They were in a remission and hospitalized for the purposes of definite diagnosis or for the performance of examination before introduction of a treatment. They were not treated with any immunomodulating or immunosuppressive drug during the last 2 years. RR-MS patients were not treated with cladribine; only SP-MS patients received the drug. The control group was recruited from patients that were admitted to the Department of Neurology due to low back pain, cervical pain, or headache; they were not treated because of any autoimmune disease nor any inflammatory disease.

The informed consent was obtained in SP-MS patients before therapy, as cladribine was used off-label. The Ethics Committee approved the experimental protocol. Demographic characteristics of patients are presented in Table 1.

Cladribine was given s.c. during 2-3 consecutive days 6 times every 6 weeks.

A total given mean cumulative dose of cladribine was 1.8 mg/kg, similarly as it was used by Rejdak et al. [22].

Venous blood was taken before the therapy and after SP-MS patients received all cycles of cladribine, i.e., after 30 weeks from baseline. The blood was allowed to clot at a room temperature. The serum was isolated by centrifugation, frozen, and stored at -70°C until laboratory assays.

### 2.2. Paraoxonase Activity

Serum PON1 activity was measured spectrophotometrically toward two synthetic (paraoxon–PON1 activity and phenyl acetate–arylesterase (AE) activity) and one natural–HTL (PON1 lactonase activity) substrates by spectrophotometric methods previously described [17, 23].

### 2.3. Homocysteine and N-Homocysteinylated Protein (N-Hcy-Protein)

Total serum Hcy was assayed by an enzyme immunoassay using a commercially available kit (Axis Shield Diagnostics Ltd., Dundee, UK) [24]. It should be emphasized that tHcy does not include Hcy bound to the *ε*-NH_2_ group of plasma proteins by an isopeptide bond (N-Hcy-protein), reflecting HTL attached to plasma proteins; therefore, measuring of this fraction of Hcy requires prior protein hydrolysis. The amount of Hcy bound to serum proteins was measured by the previously described method [24]. In brief, Hcy bound to proteins by disulphide bonds was first removed by dithiothreitol and proteins were precipitated by the addition of ethanol. Precipitated serum proteins were then hydrolysed in 6 M hydrogen chloride (HCl) at 110°C for 5 hours, and released Hcy present in the hydrolysate was measured by the method described above, respectively.

### 2.4. Antibodies against Homocysteinylated Proteins

Antigen (human N-homocysteinylated-albumin) was prepared by incubation of HTL with 10 mg/ml human albumin, in the presence of 0.2 mM ethylenediaminetetraacetic acid (EDTA) in 0.05 M potassium phosphate buffer (pH 7.4) at 37°C for 16 h.

Immunoglobulin G (IgG) antibodies against N-Hcy-albumin were measured using an in-house enzyme-linked immunosorbent assay (ELISA) [11].

### 2.5. Total Antioxidant Potential of Serum

The total antioxidant potential of the serum was assessed as the ferric-reducing ability of plasma (FRAP) method described by Benzie and Strain [25]. This method determines the ability of the antioxidant contained in the sample to reduce ferric-tripyridyltriazine (Fe^3+^-TPTZ) to a ferrous form (Fe^2+^) which absorbs light at 593 nm.

### 2.6. Statistics

The results are reported as mean ± standard deviation (SD) with the exception of PON1 activity that was shown as median (minimum–maximum). The Shapiro-Wilk test was used to test for normality. Three groups of subjects did not differ regarding age and sex; therefore, adjustment for covariates was not done and between-group comparisons were made by ANOVA. The primary aim of this study was to examine the effect of cladribine in SP-MS patients. Post- and pretreatment values were compared by the Student *t*-test for related variables (for normally distributed data) or the Wilcoxon signed rank test (for PON1 activity toward paraoxon). Repeated measures analysis of variance was used to assess the influence of treatment. *p* < 0.05 was considered significant.

## 3. Results

### 3.1. Serum PON1 Activity, Level of Hcy, N-Homocysteinylated Protein, Antibodies against Homocysteinylated Proteins, and FRAP in Untreated MS Patients (Table 2)

Serum PON1 activity toward paraoxon was not significantly different between groups; only a tendency to higher PON1 activity was observed in RR-MS patients than in SP-MS patients. Similarly, PON1 activities toward phenyl acetate and HTL did not differ between groups significantly. The distribution of PON1 genotypes was similar in all three groups (data not included). The level of Hcy was significantly higher in RR-MS patients (*p* < 0.001) and in SP-MS patients without any treatment (*p* < 0.01) in comparison to the control group (12.3 ± 5.6 *μ*M). The level of protein-bound HTL was higher in RR-MS patients (*p* < 0.05), and the level of antibodies against homocysteinylated proteins was higher in SP-MS patients (*p* < 0.05)—Table 2. Total antioxidant capacity of plasma was lower in both RR-MS and SP-MS patients (*p* < 0.001) than in control subjects but did not differ between both MS subgroups.

### 3.2. Effect of Cladribine on Serum PON1 Activity, Level of Hcy, HTL, Antibodies against Homocysteinylated Proteins, and FRAP in SP-MS Patients

**Figure 1:**
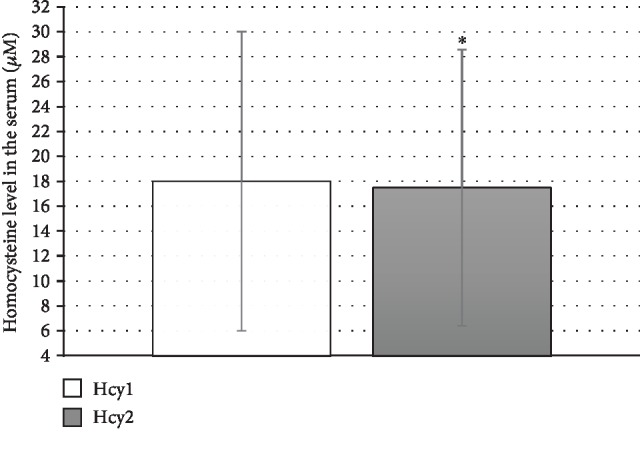
Homocysteine level before (Hcy1) and after (Hcy2) cladribine treatment in SP-MS patients; ^∗^*p* < 0.05.

**Figure 2:**
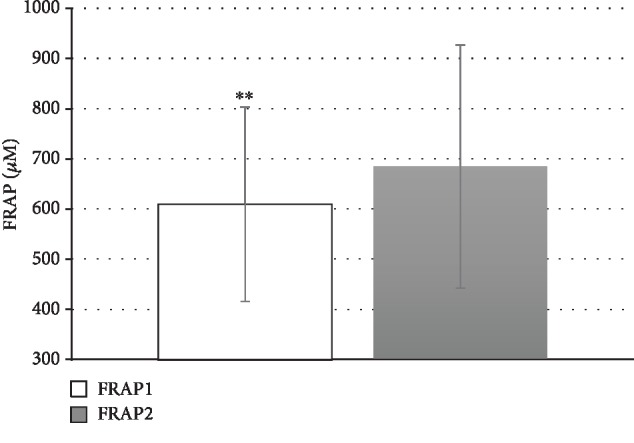
The ferric-reducing activity of plasma (FRAP) before (FRAP1) and after (FRAP2) cladribine treatment in SP-MS patients; ^∗∗^*p* < 0.01.

Cladribine treatment had no effect on PON1 activities toward paraoxon (paraoxonase activity), phenyl acetate (arylesterase activity), and HTL (lactonase activity)—Table 3. In contrast, cladribine significantly decreased the tHcy level in the serum (Figure 1) although this effect was quantitatively small, *p* < 0.05 (Table 3). Cladribine treatment reduced serum N-Hcy-protein levels; however, the difference did not achieve statistical significance. It had no effect on antibodies against homocysteinylated proteins. Cladribine significantly improved the antioxidant activity of the serum, *p* < 0.01 (Figure 2).

The neurological status of SP-MS patients treated with cladribine was stable, the EDSS score did not change after the treatment, and there was no progression of disease.

## 4. Discussion

The major findings from the present study show that in the plasma of untreated RR-MS and SP-MS patients, there is a high level of Hcy and impaired total antioxidant activity. Moreover, a high level of plasma homocysteinylated protein was observed in RR-SM patients as well as an elevated level of antibodies against homocysteinylated protein in patients with SP-MS. Cladribine in SP-MS patients has positive effects on elevated Hcy level and the total antioxidant activity of plasma but did not affect significantly PON1 activity nor protein homocysteinylation. Cladribine had also positive effects on the clinical course of disease in SP-MS patients preventing the progression of disability and stabilising the neurological status of patients.

Hyperhomocysteinemia observed in both MS groups confirms the results of the other studies conducted in MS patients [14, 15, 17]. An increased level of Hcy was also found in patients with the other neurological disorders, like dementia and Parkinson's disease [15]. It was shown that hyperhomocysteinemia is associated with cognitive impairment in MS patients [26]. A meta-analysis published in 2011 found increased levels of Hcy in MS patients and decreased levels of vitamin B12 [27]. In our study, the levels of vitamin B12 and folate were within normal limits (data not included). Another meta-analysis that searched medical databases (PubMed, MEDLINE, and Embase) for eligible studies published until June 2017 confirmed the tendency to increased levels of Hcy in MS patients compared to healthy individuals [16]. Both meta-analyses concluded that hyperhomocysteinemia can play a role in the pathogenesis of MS [16, 27]. It can be suggested that through homocysteinylation of proteins, hyperhomocysteinemia can induce immune process leading to demyelination and inflammation of the cells in the CNS of MS patients. The results of a study by Ramsaransing et al. [18] confirm that hyperhomocysteinemia is not associated with progression of MS.

In the present study, cladribine treatment had positive effects on Hcy concentration in SP-MS patients. After six courses of parenteral cladribine, the level of Hcy decreased; also, the tendency to decrease HTL level was observed, which shows positive aspect of the drug activity. However, some limitation in the results should be noticed. Despite high SD values, the effects of cladribine on Hcy were significant as assessed by *t*-test for related variables with each patient's pretreatment value serving as its own control. High variability of Hcy concentration is often observed in clinical studies. The possible role of confounding factors could not be analysed because the group was relatively small. There are many factors which affect the blood Hcy level such as age, body weight, insulin resistance, renal function, and diet (methionine as well as vitamin B6, B12, and folate content). Analysis of all these factors would require a much more number of patients. However, it is unlikely that these confounding factors changed significantly in a given patient during cladribine therapy. In our study, lower antioxidant activity of serum assessed by the FRAP method in MS patients is most probably caused by the presence of oxidative stress in MS patients that was observed in many studies [1]. Cladribine treatment resulted in an increase in the level of antioxidant capacity of serum assessed with the FRAP method in SP-MS patients. It could be explained as the protective response or compensatory mechanism to cytotoxic effects of cladribine [6]. Cladribine induces apoptosis, causes disruption of mitochondrial membrane potential, inhibits DNA repair which results in DNA strand breaks, and ultimately shifts the cell towards the necrosis pathway. Direct mitochondrial toxicity causes decreased mitochondrial membrane potential, leading to release of cytochrome C (caspase-dependent apoptosis) and nuclear translocation of apoptosis-inducing factor (caspase-independent apoptosis) [19]. It results in common side effects of cladribine treatment, i.e., lymphopenia and resulting opportunistic infections, but on the other hand, it leads also to inhibition of immune cells which results in the prevention of relapses in RR-MS patients and inhibition of MS progression in SP-MS patients [2]. It may be hypothesized that as a response to apoptosis and necrosis caused by a short-time therapy with cladribine, natural antioxidants (e.g., uric acid) are induced. Inhibition of immune response and phagocyte activity (“respiratory burst”) may also be important. Another possible explanation may be related to the fact that Hcy activates reduced nicotinamide adenine dinucleotide phosphate (NADPH) oxidase and increases the cellular production of the ROS. Consequently, a decreased level of Hcy by cladribine diminishes the release of ROS and increases FRAP [11]. It was also reported that in the experimental autoimmune encephalomyelitis (EAE), an animal model of MS, cladribine, interferes with the synaptic effects of interleukin 1 (IL-1) beta that suggests neuroprotective effects of the drug independent of its peripheral immunosuppressant action [28].

We also observed a tendency to higher PON1 activity in RR-MS patients than in SP-MS patients before treatment with cladribine. It may be associated with younger age of RR-MS patients, whereas a tendency to lower PON1 activity in SP-MS patients is probably due to older age and longer disease duration [7, 10]. Cladribine therapy did not change the activity of this enzyme in SP-MS patients. There are no other studies regarding this issue in the current literature.

## 5. Conclusions

Our study demonstrated that cladribine treatment in SP-MS patients has positive effects on the Hcy level and the total antioxidant activity of the serum. It also attenuates hyperhomocysteinemia-induced protein homocysteinylation. Hyperhomocysteinemia and lower antioxidant activity of serum (assessed by the FRAP method) were found in MS patients. It confirms the presence of oxidative stress in MS patients which was observed in many studies [1]. Also, a tendency to higher PON1 activity in RR-MS patients than in untreated SP-MS patients was observed. Cladribine therapy did not change the activity of this enzyme in SP-MS patients. The drug had positive effects on the clinical course of disease stabilising the neurological status of SP-MS patients and preventing the progression of disability. Although the observation period was quite short, treatment with cladribine can be recommended for SP-MS patients.

## Figures and Tables

**Table 1:** Characteristics of studied groups.

Variable	SP-MS patients	RR-MS patients	Control group
Number	40	30	48
Age (years)	43 ± 88^∗^	33 ± 9^###^	38 ± 11
Female sex number (%)	27 (67.5)	20 (66.6)	32 (66.6)
Disease duration from onset (years)	13 ± 8	5 ± 4^###^	N/A
EDSS score	5 ± 1	2.5 ± 1^###^	N/A

EDSS: Expanded Disability Status Scale; MS: multiple sclerosis; RR: relapsing-remitting; SP: secondary progressive. Data are expressed as mean ± standard deviation (SD), ^∗^*p* < 0.05 vs. control group; ^###^*p* < 0.001 vs. SP-MS.

**Table 2:** Serum PON1 activity, level of Hcy, HTL, antibodies against homocysteinylated proteins, and FRAP in untreated MS patients and the control group. Results are reported as mean ± standard deviation (SD) with the exception of PON1 activity (shown as median, min-max).

Variable	Untreated RR-MS	Untreated SP-MS	Control group
PON1 activity toward paraoxon (U/ml)	87.0 (56.8-368.3)	80.3 (35.0-307.1)	94.4 (44.5-369.4)
AE activity (U/ml)	114.1 ± 34.3	115.3 ± 26.7	107.8 ± 24.9
PON1 lactonase activity (U/ml)	4.8 ± 1.6	4.8 ± 1.7	4.9 ± 1.8
Hcy (*μ*M)	20.7±13.2^∗∗∗^	18.0±12.0^∗∗^	12.3 ± 5.6
HTL (*μ*M)	4.2 ± 1.4^∗^	3.5 ± 1.2	3.3 ± 1.8
Antibodies against homocysteinylated proteins (A490)	0.35 ± 0.13	0.40 ± 0.16^∗^	0.33 ± 0.12
FRAP (*μ*M)	695.4±333.9^∗∗∗^	609.8±193.7^∗∗∗^	892.0 ± 209.7

AE: arylesterase; A490: absorbance measured at 490 nm; FRAP: the ferric-reducing ability of plasma; Hcy: homocysteine; HTL: homocysteine thiolactone; PON1: paraoxonase 1; MS: multiple sclerosis; RR-MS: relapsing-remitting; SP: secondary progressive, homogenous groups, ^∗^*p* < 0.05, ^∗∗^*p* < 0.01, and ^∗∗∗^*p* < 0.001 vs. control group.

**Table 3:** Effect of cladribine on serum PON1 activity, level of Hcy, HTL, antibodies against homocysteinylated proteins, and FRAP in SP-MS patients. Results are reported as mean ± standard deviation (SD) with the exception of PON1 activity (shown as median, min-max).

Variable	SP-MS before cladribine	SP-MS after cladribine
PON1 activity (U/ml)	80.3 (35.0-307.1)	78.1 (39.3-290.0)
AE activity (U/ml)	115.3 ± 26.7	114.0 ± 27.7
PON1 lactonase activity (U/ml)	4.8 ± 1.7	4.9 ± 1.7
Hcy (*μ*M)	18.0 ± 12.0	17.5 ± 11.1^∗^
HTL (*μ*M)	3.5 ± 1.2	3.4 ± 1.2
Antibodies against homocysteinylated proteins (A490)	0.40 ± 0.16	0.39 ± 0.20
FRAP (*μ*M)	609.8 ± 193.7	685.3±242.3^∗∗^

AE: arylesterase; A490: absorbance measured at 490 nm; FRAP: ferric-reducing ability of plasma; Hcy: homocysteine; HTL: homocysteine thiolactone; MS: multiple sclerosis; PON1: paraoxonase 1; RR-MS: relapsing-remitting type of multiple sclerosis; SP: secondary progressive type of multiple sclerosis; ^∗^*p* < 0.05 and ^∗∗^*p* < 0.01 vs. untreated initial status.

## Data Availability

The data used to support the findings of this study are available from the corresponding author upon request.
